# *Blastocystis* Colonization Alters the Gut Microbiome and, in Some Cases, Promotes Faster Recovery From Induced Colitis

**DOI:** 10.3389/fmicb.2021.641483

**Published:** 2021-04-07

**Authors:** Vincent Billy, Zuzana Lhotská, Milan Jirků, Oldřiška Kadlecová, Lucia Frgelecová, Laura Wegener Parfrey, Kateřina Jirků Pomajbíková

**Affiliations:** ^1^Department of Zoology, Biodiversity Research Centre, University of British Columbia, Vancouver, BC, Canada; ^2^Institute of Parasitology, Biology Centre, Czech Academy of Sciences, České Budějovice, Czechia; ^3^Department of Medical Biology, Faculty of Science, University of South-Bohemia, České Budějovice, Czechia; ^4^Department of Pathology and Parasitology, University of Veterinary and Pharmaceutical Sciences Brno, Brno, Czechia; ^5^Department of Botany, University of British Columbia, Vancouver, BC, Canada

**Keywords:** *Blastocystis*, DNBS colitis, rat model, inflammation alleviation, gut microbiome, symbiosis

## Abstract

Protists are a normal component of mammalian intestinal ecosystems that live alongside, and interact with, bacterial microbiota. *Blastocystis*, one of the most common intestinal eukaryotes, is reported as a pathogen that causes inflammation and disease, though health consequences likely vary depending on host health, the gut ecosystem, and genetic diversity. Accumulating evidence suggests that *Blastocystis* is by and large commensal. *Blastocystis* is more common in healthy individuals than those with immune mediated diseases such as Inflammatory Bowel Diseases (IBD). *Blastocystis* presence is also associated with altered composition and higher richness of the bacterial gut microbiota. It is not clear whether *Blastocystis* directly promotes a healthy gut and microbiome or is more likely to colonize and persist in a healthy gut environment. We test this hypothesis by measuring the effect of *Blastocystis* ST3 colonization on the health and microbiota in a rat experimental model of intestinal inflammation using the haptenizing agent dinitrobenzene sulfonic acid (DNBS). We experimentally colonized rats with *Blastocystis* ST3 obtained from a healthy, asymptomatic human donor and then induced colitis after 3 weeks (short term exposure experiment) or after 13 weeks (long term exposure experiment) and compared these colonized rats to a colitis-only control group. Across experiments *Blastocystis* ST3 colonization alters microbiome composition, but not richness, and induces only mild gut inflammation but no clinical symptoms. Our results showed no effect of short-term exposure to *Blastocystis* ST3 on gut inflammation following colitis induction. In contrast, long-term *Blastocystis* exposure appears to promote a faster recovery from colitis. There was a significant reduction in inflammatory markers, pathology 2 days after colitis induction in the colonized group, and clinical scores also improved in this group. *Blastocystis* colonization resulted in a significant reduction in tumor necrosis factor alpha (TNFα) and IL-1β relative gene expression, while expression of IFNγ and IL17re/17C were elevated. We obtained similar results in a previous pilot study. We further found that bacterial richness rebounded in rats colonized by *Blastocystis* ST3. These results suggest that *Blastocystis* sp. may alter the gut ecosystem in a protective manner and promote faster recovery from disturbance.

## Introduction

*Blastocystis* is a common inhabitant of the large intestine of many animals and over a billion people worldwide (Stensvold and Clark, 2016). In developing countries, *Blastocystis* prevalence in human population can reach 84% (Poulsen et al., 2016) and 100% (El Safadi et al., 2014), and prevalence in industrialized countries ranges between 7 and 50% (Bart et al., 2013; Scanlan et al., 2014, 2016; Seyer et al., 2017; Lhotská et al., 2020). Yet the role *Blastocystis* plays in human health and disease is still unclear and the subject of ongoing debate (Lukeš et al., 2015; Chabé et al., 2017; Stensvold et al., 2020). Reported consequences of *Blastocystis* range from a pathogen that causes inflammation and disease (Jimenez-Gonzalez et al., 2012; Nourrisson et al., 2014) to a commensal or to potentially beneficial organism (Andersen and Stensvold, 2016; Stensvold et al., 2020). Until recently, the presence of *Blastocystis* in human gut was thought to be strongly associated with gastrointestinal disorders, especially irritable bowel syndrome (IBS) (Jimenez-Gonzalez et al., 2012; Poirier et al., 2012; Nourrisson et al., 2014). However, a series of reports from different parts of world have found that *Blastocystis* is more common in healthy humans (Scanlan et al., 2014; Krogsgaard et al., 2015; Seyer et al., 2017; Nieves-Ramirez et al., 2018; Mardani Kataki et al., 2019; Tito et al., 2019) than in patients with gut inflammation (Petersen et al., 2013; Krogsgaard et al., 2015, 2018; Rossen et al., 2015; Mattiucci et al., 2016; Beghini et al., 2017). A comparative study in Mexico also found that the occurrence of *Blastocystis* in healthy people was associated with lower level of fecal calprotectin, a marker of intestinal inflammation (Nieves-Ramirez et al., 2018), contradicting the expectation that *Blastocystis* is associated with, or causes, gut inflammation. The debate over whether *Blastocystis* is a pathogen or mutualist must take into account the context of the environment and hosts.

We have an incomplete, and often conflicting picture of the influence of *Blastocystis* on the mammalian immune system. Several studies reported upregulation of proinflammatory cytokines in *in vitro* and *in vivo* rodent models (Iguchi et al., 2009; Chandramathi et al., 2010; Lim et al., 2014; Ajjampur and Tan, 2016; Ajjampur et al., 2016), particularly in mice (Moe et al., 1997; Abdel-Hafeez et al., 2016; Yason et al., 2019). In rats, some inoculation studies have found no overt pathogenicity or intestinal inflammation (Defaye et al., 2018; Růžková et al., 2018), while others have documented intestinal inflammation and diarrhea following inoculation with *Blastocystis* ST1 from a symptomatic patient (Li et al., 2013). These findings often differ according to whether the *Blastocystis* isolate tested was obtained from an asymptomatic human or patient with gastrointestinal symptoms, even within subtype (Ajjampur and Tan, 2016). These findings in rodent models also suggest a complex interaction between *Blastocystis* and the host that falls in between overt pathogenicity and benign colonist. For example, Defaye et al. (2020) recently showed that *Blastocystis* ST4 from symptomatic patient induces colonic hypersensitivity and depressive-like symptoms in rats that are characteristics of IBS but did not induce gut inflammation.

*Blastocystis* is highly genetically diverse organism and has been categorized into 17 subtypes (ST) according to host specificity (Stensvold and Clark, 2016). Interestingly, its morphological uniformity coupled with a broad host specificity (e.g., nine of the subtypes have been found in humans, some of which are also common in other mammals) gave rise to the convention of describing all detected lineages as subtypes within the one genus (Stensvold et al., 2020). *Blastocystis* genomics has revealed considerable genetic diversity between subtypes, as 6 – 20% of coding genes are unique to a subtype, even in genes that probably underlie pathogenicity (Gentekaki et al., 2017). In addition, some studies found more frequent occurrence of particular subtypes of *Blastocystis* in disease, for example ST1 and ST4 in individuals with active IBS (Yakoob et al., 2010; Poirier et al., 2012; Nourrisson et al., 2014), however, some subtypes (such as ST3) occur mainly in healthy individuals (Stensvold and Clark, 2016).

*Blastocystis* colonization is associated with an altered gut bacterial microbiome, which may influence health outcomes, though the direction of causality is unknown. The gut microbiome plays an important role in immune regulation and the development of immune mediated disease (Round and Palm, 2018; Fitzgibbon and Mills, 2020). The presence of *Blastocystis* is often associated with elevated bacterial diversity and significant differences in the bacterial gut microbiome in healthy individuals (Andersen and Stensvold, 2016; Audebert et al., 2016; Krogsgaard et al., 2018; Nieves-Ramirez et al., 2018; Gabrielli et al., 2020), but not always as in some IBS patients correlates with lower richness of some protective bacteria (Nourrisson et al., 2014). Many studies also show strong differences in gut microbiome composition, with communities found in association with *Blastocystis* often reported as characteristic of a healthy gut environment, with taxa such as enriched *Prevotella* and depleted Bacteroidetes (Andersen and Stensvold, 2016), or enriched *Clostridia* and depleted Enterobacteriaceae (Audebert et al., 2016). However, particular taxa and patterns differ from study to study. These correlational studies cannot determine whether differences in the gut microbiome are a cause or consequence of *Blastocystis* colonization. So far, only one study did directly test the impact of *Blastocystis* subtype 7 (ST7) on gut microbiota in *in vivo* and *in vitro* model, and they report elevated *Lactobacillus* and *Prevotella*, but no effect on Bacteroidetes (Yason et al., 2019).

Much of the experimental work on *Blastocystis* to date utilizes clinical isolates from symptomatic human patients, which may influence findings. For example, *Blastocystis* ST7 isolated from a patient with diarrhea was recently used to test the interaction between *Blastocystis* and gut microbes (Yason et al., 2019). Li et al. (2013) obtained the isolate ST1 from a symptomatic patient. In contrast, some other authors used isolates of *Blastocystis* from asymptomatic human donors to optimize the experimental rat models, for subtypes ST1, ST3 and ST4 (Li et al., 2013; Defaye et al., 2018; Růžková et al., 2018). Nevertheless, these models allow long-term monitoring of the interactions between *Blastocystis*, the host immune system and intestinal microbiome in a model of commensal colonization.

This inconsistent view of *Blastocystis* in health and disease is a reflection of persistent gaps in the knowledge about factors influencing host colonization and the interactions between *Blastocystis* and the mammalian gut and the gut microbiome. Here we directly test the impact of *Blastocystis* ST3 colonization on the immune system and gut ecosystem alone and in combination with chemically induced gut inflammation in a rat model. Specifically, we test whether *Blastocystis* colonization induces proinflammatory cytokines, and whether *Blastocystis* protects against, or exacerbates, intestinal inflammation following chemically induced colitis. We also investigate the impact of *Blastocystis* colonization on the gut microbiome and test the hypotheses that *Blastocystis* induces higher diversity in the gut microbiome and alters gut microbiome composition. We experimentally inoculated rats with *Blastocystis* ST3 and then induced colitis after 3 weeks (short-term exposure experiment) and after 3 months (long-term exposure experiment) of colonization. Subsequently, we monitored the intensity of inflammation in colonized rats compared to the control group based on some cytokines‘ gene expressions, macroscopic and microscopic observations, clinical data, and the bacterial microbiome.

## Materials and Methods

### Animal Use and Ethical Standards

Experiments were carried out with outbred female Wistar rats obtained when 13 weeks old and 180–220 g from Envigo RMS B.V. (Horst, Netherlands; the supplier Anlab s.r.o., Prague, Czechia). All rats were housed under a controlled condition (22°C, 12:12-h light–dark cycle), provided unlimited access to autoclaved rat chow and water as well as animal health status was visually inspected at regular 24-h intervals during daily routine. Animals were always acclimated to animal facility conditions prior to start of each experiment. All rats were euthanized by cervical dislocation at the end of the experiment.

This study was carried out in the strict accordance with the recommendations in the Czech legislation (Act No. 166/1999 Coll., on veterinary care and on change of some related laws, and Act No. 246/1992 Coll., on the protection of animals against cruelty) as well as the legislation of European Union. The present experiments and protocols were approved by the Committee on the Ethics of Animal Experiments of the Biology Centre of the Czech Academy of Sciences (České Budějovice, Czechia, permit no. 33/2018) and by the Resort Committee of the Czech Academy of Sciences (Prague, Czechia).

All rats were held in individually ventilated isolator cages with HEPA filters for filtration of incoming air (Individually Ventilated Cages machine SealSafe 1291H, Techniplast s.p.a., Buguggiate, Italy; the supplier: Trigon Plus a.s., Čestlice, Czechia).

### Colonization, Experimental Setup, and Colitis Induction

To colonize experimental rats, we used the *Blastocystis*-rat model system established in our previous study (Růžková et al., 2018). Briefly, we prepared doses containing *Blastocystis* ST3 to inoculate one group of rats (i.e., colonized rats) from asymptomatic human donor stool sample using a sucrose gradient. We obtained infection doses with 10^2^–10^3^ of cysts and residuum of bacteria from donor stool sample that was not possible to remove. Subsequently, all rats were orally inoculated using an esophageal gavage. The control group (i.e., non-colonized rats) was inoculated with placebo (sterile phosphate-buffered saline – PBS). Successful colonization was confirmed by xenic culture in Jones’ medium between 16- and 25-day *post inoculation* (*p.i.*). When colonization was confirmed, the experiment continued.

**FIGURE 1:**
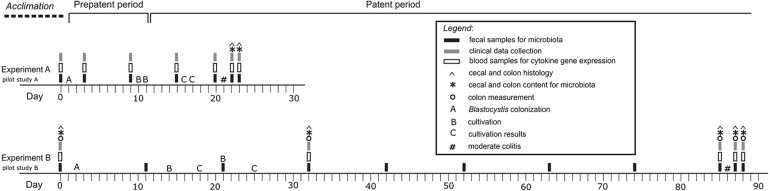
Graphical design of the experiments and pilot studies. Experiment A – the short-term *Blastocystis* ST3 colonization (i.e., 3 weeks); Experiment B – the long-term *Blastocystis* ST3 colonization (i.e., 13 weeks).

In this study, we tested the effect of the short-term and long-term *Blastocystis* ST3 colonization on the intestinal inflammation in the rat model. In both cases, we performed the pilot studies (Pilot study A and B), the designs of which corresponded to the following experiments (Figure 1). Colitis was always induced after a certain period of exposure of rats to *Blastocystis* ST3 such as 3 weeks in case of the short-term colonization (Figure 1A), and 13 weeks (i.e., 3 months) in case of the long-term colonization (Figure 1B). In both, we induced the moderate model of colitis in the patent period of colonization (i.e., rats excreted cysts in feces). The colitis was activated by the dinitrobenzene sulfonic acid (DNBS; Sigma Aldrich, St. Louis, MO, United States) (Wallace et al., 1995; Jirků-Pomajbíková et al., 2018), which was installed per-rectally (using 10-cm long catheter with 3.3 mm in diameter; type Nelaton, Dhalhausen s.r.o., Kuřim, Czechia). All rats were anesthetized with isoflurane (Forane^®^ 100 mL, Abb Vie s.r.o., Prague, Czechia) using anesthesia equipment (Oxygen Concentrator JAY-10-1.4, Longfian Scitech Co. LTD., Baoding, China; Calibrated Vaporizer Martx VIP 3000, Midmark, Dayton, OH, United States).

Each experiment included two treatment groups with balanced numbers of rats: (i) control group with colitis only (Experiment A: *n* = 10, Pilot study A: *n* = 6; Experiment B: *n* = 13; Pilot study B: *n* = 3); and (ii) group of *Blastocystis*-colonized rats with colitis (Experiment A: *n* = 10, Pilot study A: *n* = 6; Experiment B: *n* = 10; Pilot study B: *n* = 3). In case of the Experiment B (long-term exposure), we sacrificed the rat throughout the experiment running (on Day 0, 31, 85, 87, and 88) for histological analyses. In the short-term one, we collected samples for histology after colitis only. For purposes of microbial analyses, rats were randomly assigned to experimental treatment cages in pairs, taking care to change cage mater compared to the initial week-long acclimatization period to minimize microbial similarity within a cage.

### Collection of Samples, Clinical Activity, and Histology

For the purpose of this study, we used the protocols for sampling, storing and processing of samples as well as for colitis quantification according to our previous study Jirků-Pomajbíková et al. (2018). In brief, we collected samples of the blood, feces, swab samples from cecum and colon, histology samples as well as clinical data (for details see Figure 1). Histology and swab samples were collected only during the dissections in the end of experiment (Experiment A/short-term colonization) or during experiment (Experiment B/long-term colonization). Collection of blood samples and clinical data were performed while rats were anesthetized as described above. In case of blood samples, 150–200 μl were drawn from ocular plexus of rats, added into 0.5 mL EDTA tubes (Minicollect^®^, Greiner Bio-one GmbH, Kremsmünster, Austria) and processed for RNA isolation and, subsequently, for cytokine gene expression (for details see Jirků-Pomajbíková et al., 2018). Fecal samples were also collected by transferring of fecal pellets or swab samples (in case of diarrhea) to 1.5 mL microcentrifuge tubes. Then, placed to −20°C until DNA extraction.

Colitis was quantified using clinical parameters collected in the blind fashion – weight loss, stool consistency, hematochezia and hindgut length. Changes in weight were assessed using percent weight loss calculated compare with the weight on the day before colitis induction. Percent weight change was compared between treatment groups on each day with Welch’s *t*-tests followed by Benjamini–Hochberg corrections to an α value of 0.05. Analyses were conducted in Rstudio version 3.6.2 (R Core Team, 2013) and visualized using GraphPad Prism6 (GraphPad Software, San Diego, CA, United States).

Stool consistency was evaluated semi-quantitatively using scale 1 to 5, while the grade 5 corresponds to normal consistency of feces of healthy animals and grade 1 to watery diarrhea (for more details see Jirků-Pomajbíková et al., 2018). Hematochezia was assessed visually whether or not blood was present in feces (yes/no). The hindgut length was evaluated by measurement during dissections. We also quantitatively observed other clinical signs of colitis including apathy and dull coat.

Tissue hindgut samples were collected for histopathological examination, fixed in buffered 10% neutral formalin, dehydrated, embedded in paraffin wax, sectioned on a microtome (HM 430 Sliding Microtome, Thermo Shandon Limited, United States) at a thickness of 4 μm, and stained with hematoxylin and eosin (HE). All samples were observed using light microscopy (OLYMPUS BX51, Olympus Corporation, Japan).

### Analyses of Cytokine Relative Gene Expressions

Total RNA from blood samples was extracted using HybridR Blood RNA kit (GeneAll Biotechnology, Seoul, South Korea), then reverse transcribed using High Capacity RNA-to-cDNA Kit (Thermo Fisher Scientific, Waltham, MA, United States). Real-time PCR reactions were prepared using master-mix HOT FIREPol^®^ Probe qPCR Mix Plus (Solis Biodyne, Tartu, Estonia). Cytokine expressions were measured using the TaqMan gene expression assays for rats with specific primers and probes spanning exons, all obtained from Thermo Fisher Scientific: tumor necrosis factor (TNFα; amplicon length – 92 bp), receptor of interleukin 1b (IL1β; amplicon length – 74 bp), interferon gamma (IFNγ; amplicon length – 91 bp), interleukin 17re (IL17re/IL17C; amplicon length – 153 bp) and interleukin 25 (IL25; amplicon length – 80 bp) as well as ubiquitin C as housekeeping gene (UBC; amplicon length – 73 bp). For qPCR analysis was used a Light Cycler LC480 (Roche, Basel, Switzerland) and relative gene expressions of cytokines was normalized to UBC using mathematical model of Pfaffl (2001). Normalized Ct values were compared between experimental and control animals on each day by Welch’s tests followed by Benjamini–Hochberg correction to an α value of 0.05. Maximum normalization was used for graphical visualization of cytokines’ relative expressions for better illustrations. Analyses conducted in Rstudio version 3.6.2 (R Core Team, 2013) and visualized using GraphPad Prism6.

### Microbial DNA Extraction, Amplification, and Analyses

We characterized the bacterial diversity of rat gut in a total of 433 samples (fecal, colon, and cecum samples) from four experiments (Figure 1). In addition, we characterized the bacterial diversity within infectious doses used to inoculate rats with *Blastocystis*. Total fecal DNA was extracted using PSP^®^ SPIN Stool DNA Plus Kit (Stratec Biomedical, Birkenfeld Germany) according to the manufacturer’s protocol.

Library preparation and sequencing were carried out in two batches. In the first batch, we amplified samples from Pilot study A (which examined the influence of *Blastocystis* ST3 on the microbiome but did not include colitis treatment) on one 96-well plate with samples randomized across the plate and including one PCR negative control. In a second batch, 368 samples from Experiment A, Experiment B and Pilot B were amplified. Sample positions were randomized across four 96-well plates, along with four PCR negative controls per plate. We amplified the V4 region of the 16s SSU rRNA gene using the Earth Microbiome Project protocol^1^. Amplifications were performed in a 25 μL reaction volume that consisted 0.5 μL of 515F barcoded forward primer (5′-GTGYCAGCMGCCGCGGTAA-3′) and 0.5 μL of 806R reverse primer (5′-GGACTACNVGGGTWTCTAAT-3′), 1 μL of DNA, 10 μL of Phusion High-Fidelity PCR Master Mix (Thermo Fisher Scientific) and 13 μL of HyClone^TM^ Water (Thermo Fisher Scientific). PCR cycling included an initial denaturation step of 94°C for 3 min, followed by 25 cycles of 94°C for 45 s, 50°C for 60 s, 72°C for 90 s, and a final elongation step at 72°C for 10 min. PCR products were checked by gel electrophoresis then quantified in Corning^®^ 96-Well Solid Plates (VWR) using Quant-iT^TM^ PicoGreen^TM^ dsDNA Assay kit (Thermo Fisher Scientific) according to the manufacturer’s protocol. PCR products were pooled together in equal amounts (40 ng) and purified using the QIAquick PCR Purification Kit (Qiagen). Two amplicon pools were obtained: one pool for the first batch and one for the second batch. Amplicon pools were sent for sequencing at Integrated Microbiome Resource at Dalhousie University, Canada. The pools were sequenced on an Illumina MiSeq platform with 2 × 250 kit according to established protocols (Comeau et al., 2017). The raw data and metadata are deposited in the European Nucleotide Archive database^2^ under the accession number PRJEB42752.

### Sequence Processing

Post-sequencing reads were demultiplexed using idemp (Wu, 2014). We then used the DADA2 pipeline to process reads (version 1.16; Callahan et al., 2016). First, we filtered, trimmed and merged reads. We then removed chimeras. Finally, we assigned the taxonomy using the SILVA database (v. 132; Quast et al., 2013). We demultiplexed sequencing reads and processed the two batches (see above) with DADA2 separately. For the first batch, the DADA2 pipeline resulted in 1470 ASVs (Amplicon Sequence Variants) in 59 samples (+1 PCR negative control). For the second batch, the DADA2 pipeline resulted in 2367 ASVs for 368 samples (+16 PCR controls). We then removed ASVs assigned to mitochondria, chloroplasts and *Blastocystis* and other eukaryotes. ASVs that were represented by fewer than 250 reads were removed. ASVs that were present in two or fewer reads per sample were removed on a per sample basis to minimize any effects of barcode switching. We sequenced samples of the infectious dose of *Blastocystis* for each experiment. Infectious doses were isolated from donor fecal samples and cleaned as described above, but we could not remove all bacteria. We bioinformatically removed ASVs that were present in the infectious dose, including ASVs present in rats before *Blastocystis* ST3 colonization, from each experiment separately. After filtration, the dataset consisted of 775 ASVs for Experiment A, 764 ASVs for Experiment B, 593 ASVs for pilot A, and 387 ASVs for pilot B; some of the ASVs were present in multiple experiments. We constructed initial NMDS analysis and taxa summary plots to visually inspect for contamination from lab sources or switching of DNA among wells. We did not detect lab contamination but did identify three samples that were likely accidentally switched. These sample pairs were nearly identical in composition and next to one another on the DNA plates. These three samples (1101C, 1371, and 1379) were removed from the analysis. Finally, we removed samples with fewer than 5000 reads (10 samples for Experiment A and B, 7 samples for Pilot study A and 1 sample for Pilot study B). The number of samples retained for analyses for each experiment are presented in Table 2 by treatment, sample type, and day.

We assessed the microbial diversity and community composition in each experiment separately. Samples were rarefied at 5000 reads/sample for diversity analyses. For alpha-diversity, we calculated the Shannon diversity index, which measures richness and evenness, for each sample and compared groups with Welch’s test. Observed richness and Pielou’s evenness were also calculated and showed the same trends as the Shannon index, thus we present only the Shannon index for simplicity. For beta-diversity, we calculated the Bray–Curtis dissimilarity index (which takes abundance into account) and the Jaccard index (presence/absence) and compared groups with PERMANOVA. Differences in beta-diversity were visualized with NMDS plots of Bray–Curtis dissimilarity. We compared control versus treatment groups at each day for each sample type, and used a Benjamini–Hochberg *p*-value correction. Analyses were performed on Rstudio (R version 3.6.2) and scripts used for the analyses are available on the Parfrey lab Github.

To identify any taxa that are consistently enriched or depleted following *Blastocystis* colonization we used Linear discriminant analysis Effect Size (LEfSe) to identify differentially abundant bacterial taxa between groups (Segata et al., 2011) using the Galaxy/Hutlab website^3^ (accessed January 28). LEfSe analyses were performed at six taxonomic levels from phylum to genus. LEfSe analyses were conducted on single days, and analyses were also run on combined days (“Before *Blastocystis*,” “After *Blastocystis*,” and “After Colitis”) to increase our power to detect consistently enriched or depleted taxa. All analyses were run on single sample types (fecal, colon, or cecum). All LEfSe cladograms from both experiments and pilot studies are displayed in Supplementary Figures 1–4.

## Results

### Impact of the Short-Term *Blastocystis* ST3 Colonization on Colitis

**FIGURE 2:**
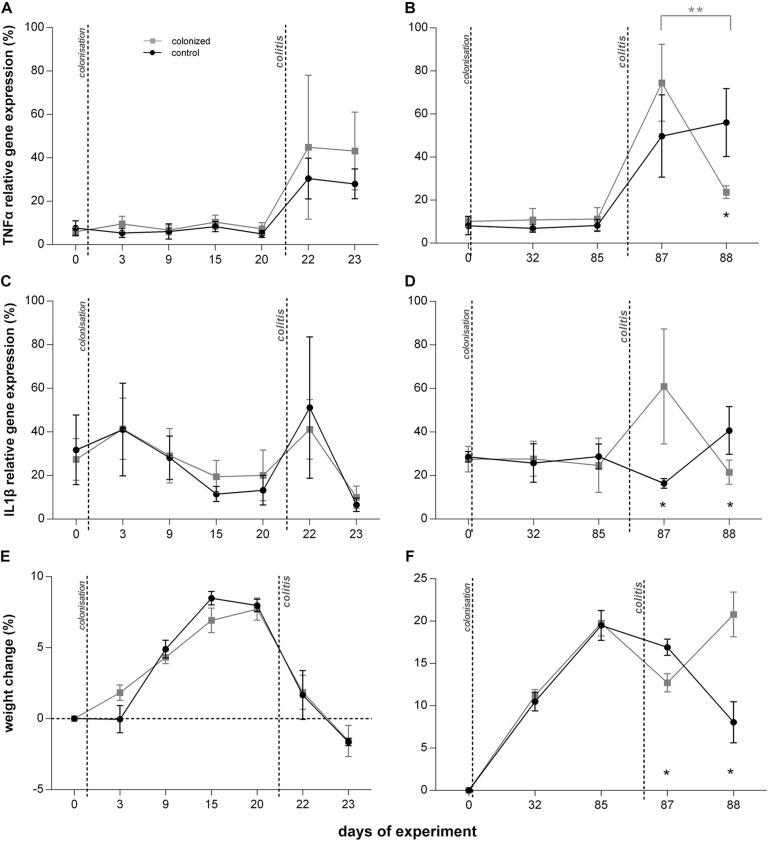
Effect of *Blastocystis* ST3 on the moderate colitis in the rat model after the short-term (3 weeks; **A,C,D**) and long-term (13 weeks; **B,D,F**) colonization. *Blastocystis*-colonized group is in gray squares, control group (i.e., non-colonized rats) in black dots. **(A,B)** TNFα gene expression relative to UBC housekeeping gene – **(A)** during short-term colonization; **(B)** during long-term colonization; **(C,D)** IL1β gene expression relative to UBC housekeeping gene – **(C)** during short-term colonization, and **(D)** during long-term colonization; **(E,F)** % weight changes after colitis induction (calculated by comparing at Day 0) – **(E)** during short-term colonization; **(F)** during long-term colonization. Differences between groups calculated with Welch’s *t*-tests followed by Benjamini–Hochberg correction. Error bars are standard errors; *P = 0.05–0.01, **P = 0.01–0.001, ***P < 0.001.

**FIGURE 3:**
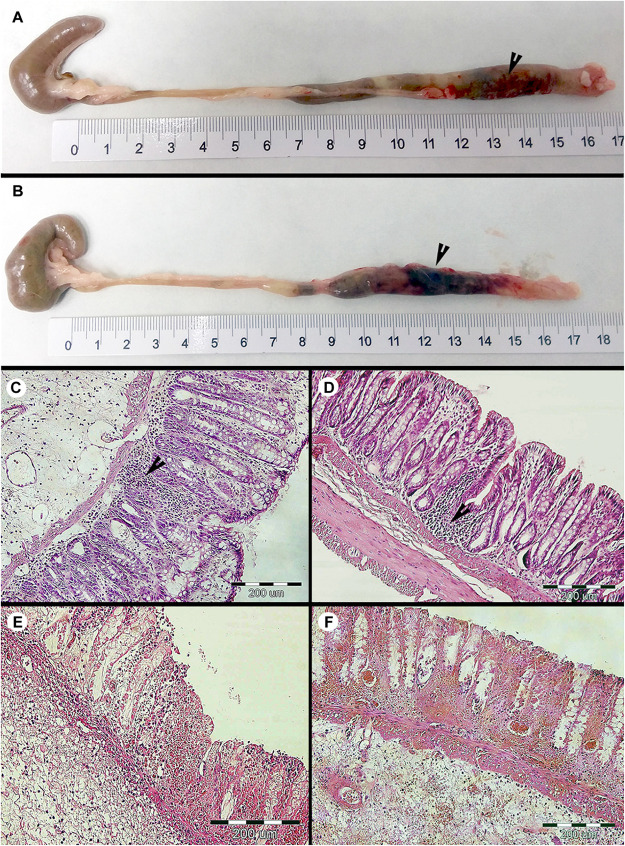
Macroscopic and microscopic evaluation of the large intestine of the colonized and non-colonized rats after the DNBS moderate colitis during the short-term *Blastocystis* ST3 colonization (i.e., 3 weeks). **(A)** intestine of non-colonized rats with the colitis; **(B)** intestine of colonized rats with the colitis (black arrows indicate the inflammation in the large intestine). The histological cross sections of the rat large intestine after induction of colitis: **(C,D)** with inflammatory-cell infiltration – **(C)** non-colonized rats and **(D)** colonized rats (black arrows indicate inflammatory infiltrate in mucosa), and **(E,F)** foci with segmental mucosal necrosis – **(E)** non-colonized rats and **(F)** colonized rats. Histological examination was not conducted prior to colitis induction in the short-term experiment and macroscopically the intestine appeared healthy with the length in average 19 cm.

We found that short-term colonization of *Blastocystis* ST3 (Figure 1, Experiment A) had no effect on the manifestation of colitis in a rat model. After colitis induction all rats developed symptoms including diarrhea and hematochezia, apathy and dull coat (Table 1), and weight loss (Figure 2E). No differences in the severity of intestinal inflammation were found between colonized and non-colonized rats based on measurements of TNFα and IL1β relative gene expression (Figures 2A,C), weight (Figure 2E), clinical data (Table 1), and macroscopic observation of the intestine (the length of inflamed intestine was in average 16cm after colitis induction in both groups; Figures 3A,B). Histological examination of the colon revealed no difference in inflammation between both groups (Figures 3C,D). We observed areas of chronic inflammatory infiltrate of various intensity with lymphocytes and plasma cells, with an admixture of eosinophils and macrophages. The infiltrate was present throughout various layers of mucosa. Other findings were goblet cell hyperplasia, hyperemia, minor focal hemorrhage, reactive hyperplasia of lymphatic submucosal tissue, submucosal edema, and focal mild fibrosis of the mucosal surface. In some areas of the mucosa, there were foci of segmental to diffuse mucosal necrosis with neutrophilic inflammatory reactions within necrotic tissue and hemorrhages (Figures 3E,F). We did not detect *Blastocystis* cells in the colonic tissues.

**TABLE 1:** Clinical response to colitis with and without *Blastocystis* ST3 after short-term and long-term colonization.

Experiment	Days p.i.*	Days *p.c.*	Number of individuals**		% with hematochezia		Mean fecal consistency	
			Control	Colonized	Control	Colonized	Control	Colonized
Short-term colonization	−1	−21	10	10	0	0	4,9	4,9
	3	−18	10	10	0	0	5	5
	9	−12	10	9	0	0	5	5
	15	−6	10	9	0	0	5	4,9
	20	−1	10	9	0	0	4,9	5
	22	1	5	5	100	100	1,8	1,6
	23	2	5	4	100	100	1	1
Long-term colonization	−1	−85	13	16	0	0	4,9	4,8
	32	−54	12	10	0	0	5	5
	85	−1	10	9	0	0	5	5
	87	1	4	4	100	100	2	1
	88	2	4	4	100	100**^#^**	1	3

Data were collected in double blind fashion. (p.i., post inoculation; p.c., post colitis). *Indicates days before and post inoculation with Blastocystis. **Decreasing number of rats over course of experiment is due to negativity of a few animal for Blastocystis after inoculation and due to sacrification for collection of samples for histology. **^#^**Hematochezia was less intensive compared to control group on same day (Day 88) and compared to colonized rats day earlier (Day 87).

**FIGURE 4:**
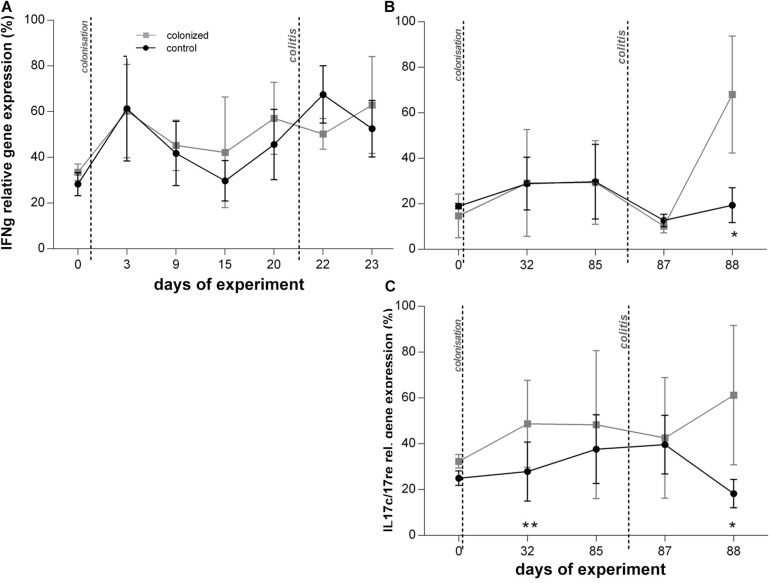
Graphical visualization of the relative gene expression of other measured cytokines over the course of the *Blastocystis* ST3 colonization and colitis induction. *Blastocystis* colonized group is in gray squares, control group in black dots. All cytokines are measured to UBC housekeeping gene. **(A,B)** IFNγ gene expression relative – **(A)** during the short-term colonization (3 weeks), **(B)** during the long-term colonization (13 weeks); **(C)** IL17re/IL17C gene expression relative during the long-term colonization only. Differences between groups calculated with Welch’s *t*-tests followed by Benjamini–Hochberg correction. Error bars are standard errors; *P = 0.05–0.01, **P = 0.01–0.001. ***P < 0.001.

Prior to colitis, there were no differences in clinical measures or the relative expression of cytokines between colonized and control groups (Table 1 and Figures 2–4). Histological examination was not conducted prior to colitis induction in this experiment and macroscopically the intestine appeared healthy with the length in average 19 cm. Expression of IFNγ was similar across groups (Figure 4A), and TNFα gene expression did not differ significantly across treatment groups (Figure 2A).

We attempted to measure IL17re/IL17C and IL25 during the short-term exposure experiment, but expression levels were at or below detection thresholds and did not yield usable data.

### Impact of the Long-Term *Blastocystis* ST3 Colonization on Colitis

Long term *Blastocystis* ST3 colonization altered the manifestation of induced colitis in rats. The *Blastocystis* colonized group initially had more severe colitis on the first day post colitis, but a significant reduction in inflammatory cytokines and clinical symptoms 2 days post colitis indicate faster recovery. Again, we attempted to measure also IL25, but no usable data as mentioned above.

**FIGURE 5:**
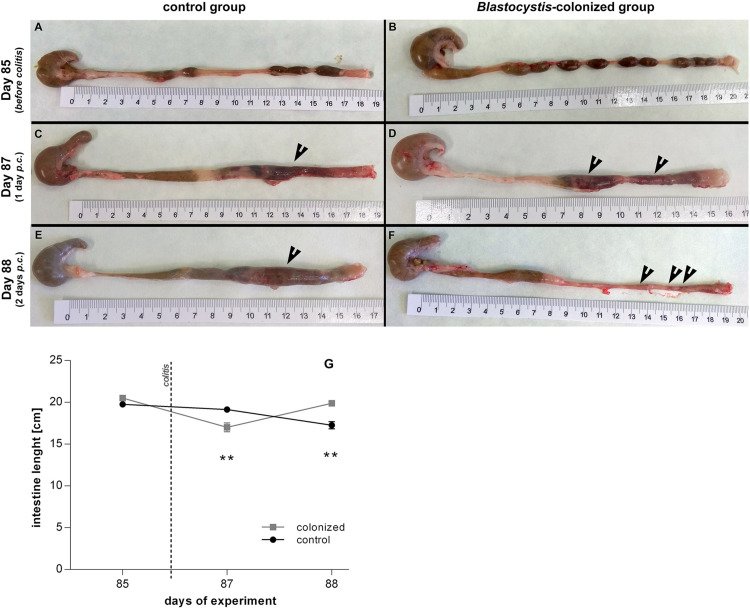
Macroscopic evaluation of the intestine length between *Blastocystis* ST3-colonized and non-colonized (i.e., control) rats, also before and after the induction of moderate DNBS colitis induction. **(A,B)** Prior to induction of colitis (Day 85), without gut inflammation, the length of the intestine ranged between 19.5 to 20.5 cm in both groups – **(A)** intestine of non-colonized rats; **(B)** intestine of colonized rats. **(C–F)** After induction of colitis (Day 87 and 88), there were differences in the length of inflamed intestine between groups and days after colitis – **(C,E)** inflamed intestine of non-colonized rats was reduced to average 18–19 cm on Day 87 and to average 16 cm on Day 88 (black arrows indicate inflammation in the large intestine); **(D,F)** inflamed intestine of *Blastocystis*-colonized rats was reduced to average 16 cm on Day 87, while on Day 88 the intestine extends almost to the original length (i.e., approximately 19.5 to 20 cm; black arrows indicate inflammation in the large intestine). **(G)** Graphical visualization of the changes in the intestine length in both groups of rats over the course of colitis (Day 85, 87, 88; colonized rats are in gray squares, non-colonized in black dots). Differences between groups calculated with Welch’s *t*-tests followed by Benjamini–Hochberg correction. Error bars are standard errors; *P = 0.05–0.01, **P = 0.01–0.001. ***P < 0.001.

**FIGURE 6:**
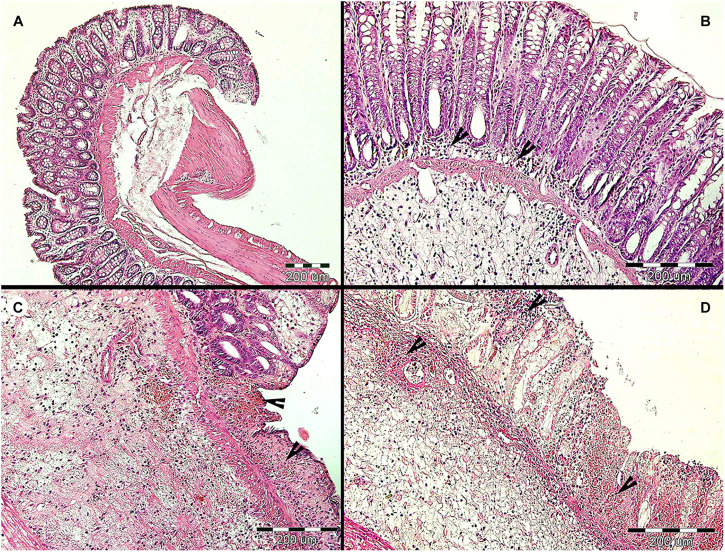
Histopathological examination of the large intestine of rats during the long-term *Blastocystis* ST3 colonization and after the DNBS colitis induction. **(A)** Representative cross section of the large intestine of the control rats (i.e., non-colonized) before colitis induction on Day 85 (similar results were found on Day 32 which is not displayed here). **(B)** Representative cross section of the large intestine of the colonized rats before colitis; there was found the moderate lymphoplasmacytic inflammation (indicated by black arrows) on Days 32 and 85. **(C,D)** representative cross sections of the large intestine from control **(C)** and colonized **(D)** rats after colitis induction; there was revealed the chronic ulcerative colitis (indicated by black arrows) in both groups on Days 87 and 88 while the second day post colitis induction (Day 88) in colonized rats the inflammation was macroscopically milder and more localized (for details see Figure 5).

Before colitis induction, inflammatory cytokines and clinical markers are similar between *Blastocystis*-colonized and control group, though we detect a few mild indicators of intestinal inflammation in colonized rats. In the absence of colitis, most cytokines, including TNFα, and clinical measures show no difference between both experimental groups (Figures 2B,D,F and Table 1). We find that relative gene expression of the cytokine IL17re/IL17C was slightly, but significantly, increased in colonized rats at Day 32, but it did not differ at later timepoints (Day 85; Figure 4C). Macroscopic evaluation of the hind-gut length shows no differences between both groups (Figures 5A,B,G). Histological examination of the cecum and colon before colitis (Day 32 and 85) revealed no pathomorphological changes in control rats and moderate chronic lymphoplasmacytic colitis in *Blastocystis*-colonized rats (Figures 6A,B). Rats in both groups were in good health status and had well-formed stools.

One day after colitis induction (Day 87; Figure 1, Experiment B), we found elevated markers of inflammation and worse health indicators of rats colonized with *Blastocystis* ST3 compared to the control group, and these trends were reversed 2 days post colitis induction (Day 88). On Day 87, *Blastocystis*-colonized rats showed more intense intestinal inflammation exhibited by a trend toward higher TNFα gene expression (Figure 2B), significantly elevated IL1β relative gene expression (Figure 2D), significantly greater weight loss (Figure 2F) and shortened intestines (Figure 5G). Elevated inflammation was also observed macroscopically in the large intestine (Figures 5C–F). Clinical data showed the similar results in both groups after colitis on Day 87 and also on Day 88 (Table 1). We observed apathy and dull coat after colitis induction in both groups.

Interestingly, the second day post colitis (Day 88; Figure 1, Experiment B), the inflammatory response in *Blastocystis*-colonized versus control rats changes considerably in comparison with previous day (i.e., 1 day post colitis – Day 87). Here, we observed a significant reduction in intestinal inflammation in colonized rats compared to the control group. Colonized rats showed significantly lower level of the gene expression of pro-inflammatory cytokines TNFα and IL1β in colonized group (Figures 2B,D) and also significant weight gain (Figure 2F). The level of relative gene expression for both IL17re/IL17C and IFNγ (Figures 4B,C) was similar between groups at Day 87 but was significantly elevated in colonized rats on Day 88. Macroscopically, the large intestine of colonized rats demonstrated a reduction in inflammation the first day after colitis, while extending to the original length of 20 cm (Figures 5E–G). No significant difference in clinical data was observed between both groups over the course of experiment (Table 1), though hematochezia in colonized rats appeared to be less intense than control rats but observed only by eye.

After colitis induction, chronic active ulcerative colitis of various intensity using histology was detected in both treatment groups (Figures 6C,D). In colonized rats on Day 88, the inflammation was macroscopically more localized (Figure 5F). We did not detect *Blastocystis* cells in the colonic tissues. In Figure 6, we demonstrate only the representative colon histological section due to colitis.

### Impact of *Blastocystis* on the Bacterial Microbiome

**FIGURE 7:**
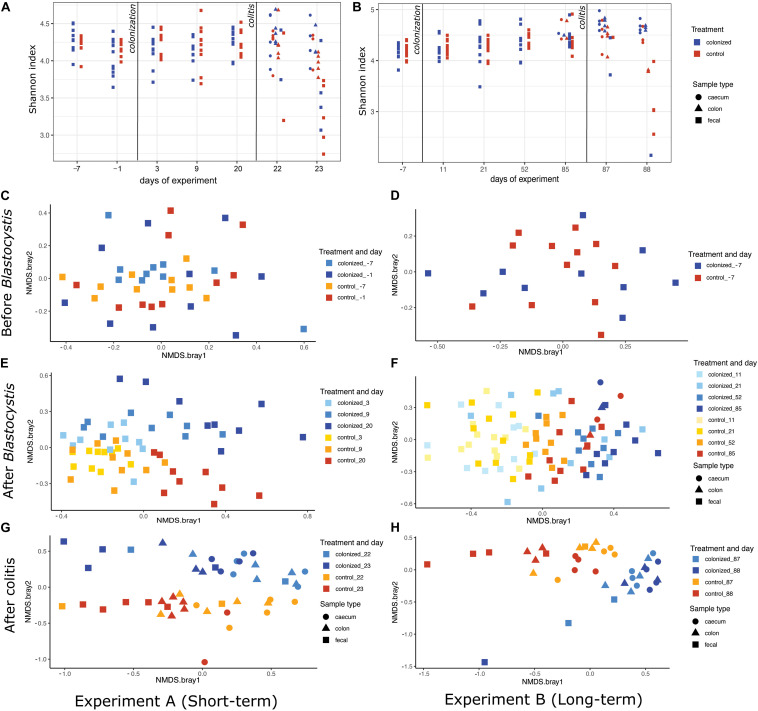
Effect of *Blastocystis* ST3 colonization on rat gut bacterial diversity before and after colitis. *Blastocystis* ST3-colonized group is in blue, control group (i.e., non-colonized rats) in orange. **(A,B)** Alpha-diversity over time measured with the Shannon index. **(A)** Short-term colonization (3 weeks). **(B)** Long-term colonization (13 weeks). Sample types are represented by shapes with cecum (circle), colon (triangle), and fecal (square). The first vertical line indicates *Blastocystis* ST3 inoculation and the second vertical line indicates the induction of colitis using DNBS. **(C–H)** NMDS plots of Bray-Curtis dissimilarity visualize the difference in gut microbiota composition between the control group (warm shades) and rats colonized by *Blastocystis* ST3 (cool shades). Prior to *Blastocystis* ST3 inoculation, fecal samples: **(C)** short-term colonization and **(D)** long-term colonization. After *Blastocystis* ST3 colonization: **(E)** short-term colonization and **(F)** long-term colonization. After colitis induction: **(G)** short-term colonization and **(H)** long-term colonization. Differences between groups are calculated with a PERMANOVA followed by Benjamini-Hochberg correction on single days and single samples type. Results of statistical tests are presented in Table 2.

We found no significant effect of *Blastocystis* ST3 colonization on the bacterial alpha diversity before colitis induction across both experiments (Figures 7A,B and Table 2). Following colitis induction, we observe higher bacterial diversity in *Blastocystis* ST3-colonized rats in the long-term colonization only (Figure 7B and Table 2). More specifically, we found higher bacterial diversity 2 days after colitis (Day 88) in colon samples (*p* = 0.013). No significant differences in alpha diversity were observed after colitis in the short-term experiment, though the trend was similar (Figure 7A and Table 2).

**TABLE 2:**
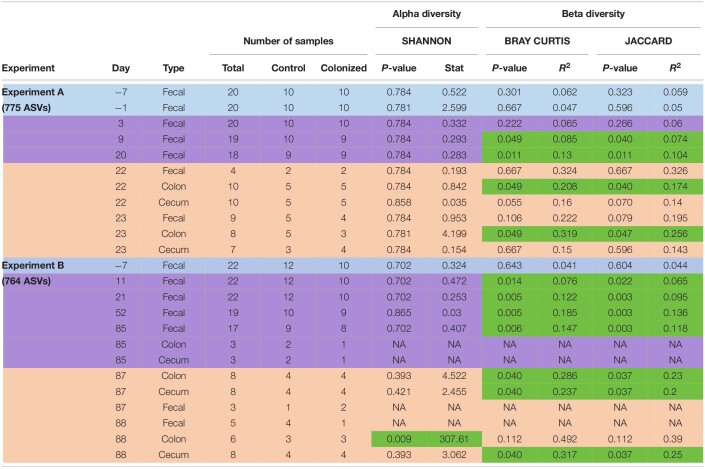
Summary of number of ASVs and samples used for analyses and results of alpha and beta diversity for both experiments (long-term and short-term colonization), from all days and sample’s types.

*In blue are the analyses before Blastocystis ST3 inoculation, in purple are the analyses after Blastocystis ST3 inoculation and in pink are the analyses after the induction of colitis. In green are the significant results. NAs mean that sample sizes were too small to run the statistical test.*

We revealed a significant effect of *Blastocystis* ST3 on the gut bacterial community composition across most days and both experiments (Figure 7 and Table 2, purple rows). Bray–Curtis and Jaccard results agree in nearly all comparisons, meaning that experimental groups differed in both the abundance and the presence/absence of bacterial taxa (Table 2). Differences in gut microbiota composition are apparent by Day 9 (short term) and Day 11 (long term) after *Blastocystis* inoculation and get stronger over time, as can be seen by the increasing amount of variation explained by treatment group (R values; Table 2). Communities do not differ on Day 3 after inoculation despite large sample sizes (Bray–Curtis *p* = 0.221, Jaccard *p* = 0.27; Table 2), perhaps because Day 3 is early in the prepatent period of *Blastocystis* colonization; the patent period begins roughly 7 to 9 days after inoculation. Similar trends of are observed in the pilot studies, with strong differences particularly apparent in the long-term colonization pilot study (pilot B) between *Blastocystis* ST3 colonized and control groups in NMDS plots of Bray–Curtis dissimilarity, though sample sizes are small (Supplementary Figure 5).

After the induction of colitis, strong differences in the community composition between *Blastocystis* ST3 colonized and control treatment groups are apparent in NMDS plots (Figures 7G,H). These differences are statistically significant for some, but not all, sample types and days (Table 2, pink rows), likely as a result of greater variation in community composition across individuals coupled with smaller sample sizes.

**FIGURE 8:**
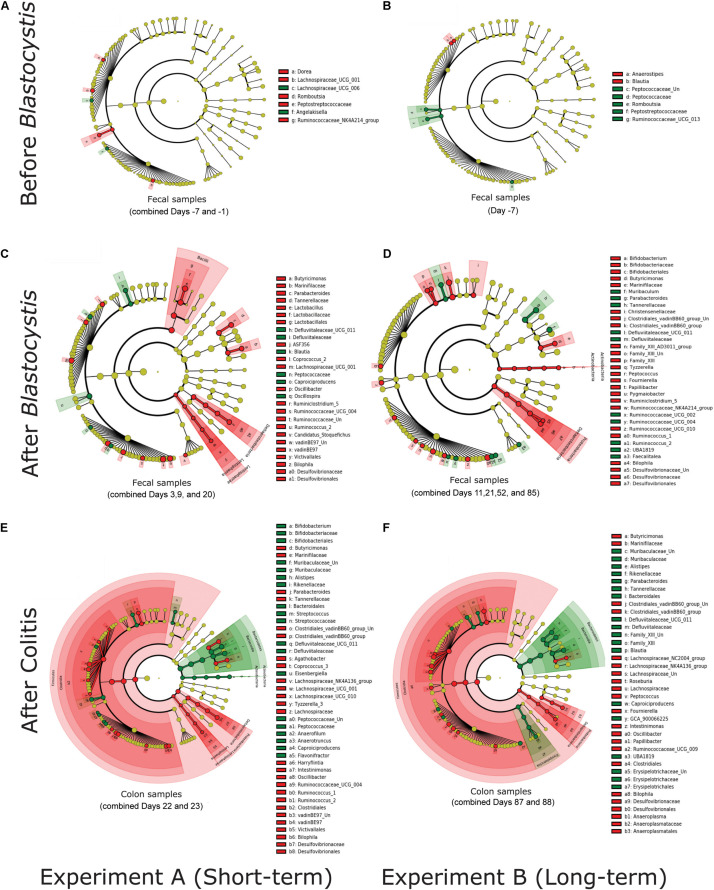
*Blastocystis* ST3 colonization alters the abundance of a varied suite of bacterial taxa before and after colitis, some of which are consistent across the short-term (3 weeks) and the long-term (13 weeks) colonization. Differential relative abundance of taxa was assessed with linear discrimination analysis of effect size (LEfSe) analysisat the phylum to the genus level. Each cladogram represents all taxa in the study. Yellow circles indicate no difference in relative abundance. Bacterial taxa significantly enriched in *Blastocystis* ST3-colonized group are in red (Kruskal-Wallis *P* < 0.05), while bacterial taxa enriched in the control are in green. **(A,B)** LEfSecladograms from rat fecal samples before *Blastocystis* colonization. **(A)** Short-term colonization with combined Days −7 and −1. **(B)** Long-term colonization with Day −7. **(C,D)** LEfSecladograms from rat fecal samples after *Blastocystis* ST3 colonization. **(C)** Short-term colonization withcombined Days 3,9, and 20. **(D)** Long-term colonization with combined Days 11, 21, 52, and 85. **(E,F)** LEfSecladograms from colon samples after colitis induction. **(E)** Short-term colonization with combined Days 22 and 23. **(F)** Long-term colonization with combined Days 87 and 88. All LEfSecladograms from singleand combined days, and all sample’s types and from both experiments and Pilot studies are presented in Supplementary Figures 1–4.

We investigated the ASVs and clades of bacteria driving differences between treatment groups. We calculated differentially abundant ASVs and clades with LEfSe on individual days and in the periods before and after *Blastocystis* inoculation, as well as after colitis induction, to assess *Blastocystis* colonization induces consistent changes in the microbiome. Before *Blastocystis* inoculation, we identified few bacterial taxa that differed between both experimental groups (Figures 8A,B and Supplementary Figures 1, 2A–D). These taxa are not differentially enriched in subsequent time points (Figures 8C–F) and likely reflect background variation across rats. After the inoculation of *Blastocystis* ST3, we identified a greater number of differentially abundant bacterial taxa (Figures 8C,D). For the most part, the differentially enriched taxa vary across experiments, even at higher taxonomic levels (Figures 8C,D and Supplementary Figures 1–4). For example, *Akkermansia* was enriched in the *Blastocystis*-colonized group across two experiments and across several days, *Izimaplasmatales* was enriched in the *Blastocystis*-colonized group in the short-term experiment, and *Parabacteroides* was depleted in the long-term colonization only (Figure 8). Some taxa are enriched in one experiment and depleted in another (Figures 8C,D). For instance, *Ruminococcus*_2 and *Parabacteroides* were depleted in the long-term colonization but enriched in the short-term colonization (Figures 8C,D), while *Bifidobacterium* was enriched in the long-term colonization but depleted in the short-term colonization (Figures 8C,D). In contrast to this variability, we identified three taxa that shift in consistent ways across both experiments and pilot studies and across days and samples types within each experiment. *Bilophila* (Deltaproteobacteria) and *Butyricimonas* (Bacteroidales) are consistently enriched in the *Blastocystis*-colonized group, while the family *Defluviitaleaceae* (Clostridiales) is consistently depleted (Figures 8C,D and Supplementary Figures 1–4).

After colitis induction, the bacterial microbiome shifts markedly in response to the associated inflammation and diarrhea. We continue to observe enrichment of *Bilophila* and *Butyricimonas* and depletion of Defluviitaleaceae along with large suite of differentially abundant taxa (Figures 8E,F). LEfSe analysis on combined days after colitis in colon samples, where sample numbers are most robust, suggests broad enrichment of the Clostridiales (Firmicutes) and depletion of Bacteroidales (Bacteroidetes) in both the short and long-term experiment (Figures 8E,F). However, this pattern varies across days and sample types (Supplementary Figures 1, 2). At the genus level, we once again observed an enrichment of the genus *Bilophila* and *Butyricimonas* and a depletion of the genus Defluviitaleaceae in *Blastocystis*-colonized group in both experiments. Amidst these strong similarities, there are still many enriched taxa differ across experiments (Figures 8E,F).

## Discussion

*Blastocystis* is one of the most common intestinal protists found in humans and animals but its role in health and effect on the microbiome remain poorly understood. While *Blastocystis* is sometimes pathogenic and associated with inflammation and gastrointestinal symptoms, an increasing number of studies show that *Blastocystis* is often present in asymptomatic people and is more common in health than in disease. To improve our understanding of the role of *Blastocystis* in health and in the gut ecosystem, we used a rat model (Růžková et al., 2018) to test the impact of commensal *Blastocystis* colonization alone and the ability of *Blastocystis* ST3 to protect against on chemically induced intestinal inflammation in an established model of Crohn‘s disease (Jirků-Pomajbíková et al., 2018). Our results suggest that *Blastocystis* ST3 is largely a benign colonizer of rats and that long term exposure to *Blastocystis* ST3 may promote recovery from induced colitis. Across all experiments, we find that *Blastocystis* ST3 alters microbiome composition but does not lead to higher bacterial diversity.

### Response to *Blastocystis* ST3 Colonization Alone

*Blastocystis* colonization alone had little impact on the host immune system and overall host health by most clinical and metrics across experiments, though we detected a mild inflammatory response during the long-term experiment. We found no significant differences in clinical measurements, such as weight change and stool consistency, between rats colonized with *Blastocystis* and controls across experiments (Figures 2–6). Similarly, we found no difference in the expression of proinflammatory cytokines TNFα and IL1β (Figure 2), or in IFNγ (Figure 4) in response to *Blastocystis* colonization across experiments. Histology results from the long-term experiment at 32 and 85 days post inoculation showed mild intestinal inflammation, characterized by a mild lymphocyte infiltrate into the mucosal layer and goblet cell hyperplasia. At Day 32, we also found elevated gene expression IL17re/IL17C. We do not detect *Blastocystis* invasion into epithelial tissue at any point before or after colitis induction. In the short-term experiment histology samples were not collected in the absence of colitis and measurements of IL17re/IL17C were below detection thresholds.

### Influence of *Blastocystis* on the Response to Induce Colitis

We found that *Blastocystis* alters the immune response to induced colitis, but only following long-term colonization. Long-term colonization with *Blastocystis* was associated with a more severe initial response to colitis induction followed by faster recovery as measured by induction of inflammatory cytokines and clinical markers. This trend toward faster recovery following colitis was also observed based on clinical observation in in a pilot study of long-term exposure (Pilot B), cytokines were not measured. However, no differences between experimental groups were observed in the short-term exposure experiment. The difference in the immune response to *Blastocystis* before and after colitis between the short- and long-term exposure experiments is surprising and potential explanations are discussed below. In both experiments, the initial inflammatory response to induced colitis (1 day post induction) included elevated gene expression of proinflammatory cytokines TNFα (Figure 2), as well as clinical markers of disease such as hematochezia and weight loss (Table 1), apathy and dull coat. Inflammation was also apparent in macroscopic and microscopic examination of the intestine for both experiments (Figures 3, 5, 6). In the short-term colonization experiment, these varied measures of inflammation were similar across experimental groups and at first day and second day post colitis. However, the time-course of the inflammatory processes was different in *Blastocystis*-colonized rats in the long-term colonization experiment.

In the long-term colonization experiment intestinal inflammation was more intense in *Blastocystis*-colonized rats compared to controls first day post colitis as measure by clinical condition of rats (Table 1), macroscopic examination of the intestine (Figure 5) and gene expression of proinflammatory cytokines TNFα and IL1β (Figure 2). Recovery at 2 days after colitis (Day 88) in *Blastocystis* colonized rats was characterized by improvement in macroscopic measures of inflammation (Figure 5) and weight gain (Figure 2), as well as changes in cytokine expression. Colonized rats had significantly lower levels of relative gene expression of the proinflammatory cytokines TNFα and IL1β compared to controls (Figure 2), as well as induction of IFNγ and cytokine IL17c/IL17re (Figure 4). Histological and clinical data were not significantly different between groups at Day 88, although we did observe a non-significant trend of less intense hematochezia and better fecal consistency.

Overall, further studies are needed to elucidate how *Blastocystis* changes the host immune response over time using more immune markers and approaches, and how it differs between its subtypes. In this study, we used an acute model of gut inflammation that lasts only 2–3 days. To more comprehensively understand the role of *Blastocystis* in intestinal inflammation, long-term models of colitis, such as severe DNBS colitis (Jirků-Pomajbíková et al., 2018) or T-cell transmission colitis (Eri et al., 2012), will need to be used in future studies.

### What Explains the Differences Between Our Results and Findings That *Blastocystis* Is Pathogenic?

Our results contrast, in part, with other studies of *Blastocystis* colonization in rodent models. Many studies have reported an association between *Blastocystis* and pathogenicity and attributed pathogenicity to factors including the status of the mucin barrier, diversity of microbiota, its effect on tight junctions related to intestinal permeability, and upregulation of proinflammatory cytokines in the gut wall (Wawrzyniak et al., 2013; Ajjampur and Tan, 2016; Ajjampur et al., 2016; Audebert et al., 2016; Lepczyńska et al., 2017; Siegwald et al., 2017; Lepczyńska and Dzika, 2019). Yet, other studies, or in some cases the same studies, in rodents report no effect of *Blastocystis* on measures of health such as weight loss, diarrhea, and cytokine expression. Variation is extensive across studies. For example, a study that inoculated rats with *Blastocystis* ST4 strain RN94-9 (a strain isolated directly from lab rats) found that colonized rats did not exhibit clinical symptoms, but expression of IFNγ and TNFα was elevated 2 or 3 weeks after inoculation (Iguchi et al., 2009). Other studies have observed that *Blastocystis* exacerbates disease (Tan et al., 2008; Ajjampur et al., 2016; Kumarasamy et al., 2017), or is associated with various immune and inflammatory responses (Chandramathi et al., 2014; Defaye et al., 2020), even in the absence of overt pathology (Iguchi et al., 2009; Defaye et al., 2020). Interpreting our results in the context of other studies on *Blastocystis* is complicated by the huge range of variation in previously reported results. The largest sources of variation are likely (i) whether the *Blastocystis* isolate is from a symptomatic or asymptomatic human donor, (ii) differences in subtype and genetic variability within subtypes, (iii) differences in model systems, including the contrast between mouse and rat models (Ajjampur and Tan, 2016), and (iv) our results suggest that the timeline of *Blastocystis* colonization is also an important determinant of the host response, and most studies to date have been short duration. We discuss these sources of variability and implications for interpreting our results.

#### Differences by Clinical Status of Donor

Differences in the immune response reported across studies may be explained in part by variation in the clinical status of the human donor of *Blastocystis*. Across studies, *Blastocystis* strains isolated from symptomatic human donors (e.g., patients with diarrhea) are more often correlated with increased pathogenicity (Ajjampur and Tan, 2016). For example, ST1 symptomatic human donor led to lethargy and intestinal inflammation, even as rats did not lose weight or develop diarrhea (Li et al., 2013). In contrast, a number of studies report that isolates from asymptomatic human donors do not result in clinical symptoms, including for *Blastocystis* ST3 (this study), *Blastocystis* ST1 (Růžková et al., 2018), and *Blastocystis* ST3 and ST4 (Defaye et al., 2018). Yet, the reported impact of *Blastocystis* varies even across studies using *Blastocystis* isolated from asymptomatic donors and at least some negative consequences of *Blastocystis* reported (e.g., Chandramathi et al., 2014; Defaye et al., 2020). Going forward, it will be useful to standardize measurement of inflammation and clinical impacts, so, that the role of *Blastocystis* genetic diversity and the context of the host and gut ecosystem can be more fully disentangled.

#### Differences in Response to *Blastocystis* by Genetic Diversity

Differences in the immune response reported across studies may be explained in part by differences in *Blastocystis* subtype as well as genetic variation within subtypes. Subtypes vary greatly in genetic diversity, including virulence factors (Gentekaki et al., 2017). Recent results in rats also show that subtypes differ in the dose required to colonize the rat with a lower dose required for ST4 to colonize rats compared to ST3 (Defaye et al., 2018), which may result from different interactions with the immune system. As in this study, Defaye et al. (2018) found *Blastocystis* ST3 and ST4 in the intestinal lumen and in close contact with epithelial cells, but no invasion into tissue. It is worth mentioning that *Blastocystis* ST3 is associated with both symptomatic and asymptomatic infections in humans (Tan et al., 2008; Wong et al., 2008; Jones et al., 2009). Kumarasamy et al. (2017) report weight loss and intestinal inflammation in rats as a result of colonization by *Blastocystis* ST3 isolated from a symptomatic patient but did not observe diarrhea. Our donor of *Blastocystis* ST3 was asymptomatic.

#### Differences Between Mouse and Rat Models

On balance, studies on *Blastocystis* colonization in mice report more severe pathology and elevated immune response in comparison to rats (Ajjampur and Tan, 2016; Růžková et al., 2018). *Blastocystis* colonization of mice with human isolates is more difficult to establish and mice are more likely to mount an immune response that prevents colonization (Ajjampur et al., 2016; Defaye et al., 2018). Previous studies interrogating the relationship between *Blastocystis* ST7 and the microbiome in mice used chemical injury to allow *Blastocystis* to establish in the gut (Yason et al., 2019), which likely has consequences for interpreting results. This disturbance, and/or the fact that the ST7 isolate is derived from a patient with diarrhea, may underlie the changes in the gut microbiome and immune environment. When colonization is established in mice, *Blastocystis* is frequently associated with clinical symptoms such as weight loss, diarrhea and lethargy, and an intense inflammatory reaction with the formation of lymphocytic infiltrate in the hindgut mucosa and submucosa (Moe et al., 1997; Elwakil and Hewedi, 2010; Ajjampur et al., 2016). Thus, it is difficult to compare the results here to previous results in mouse models. In any case, rats are a closer model to humans as both species maintain long-term colonization of *Blastocystis* without overt pathology.

#### Timeline of Colonization

Our results also reveal surprising differences between the short and long-term exposure experiments in the host response to *Blastocystis* itself and in the context of colitis. The timeline of *Blastocystis* colonization studied here is longer than most studies (but see Kumarasamy et al., 2017) and to our knowledge this is the first study to colonize animals with *Blastocystis* prior to inducing inflammation to investigate a role for *Blastocystis* in protecting against, or exacerbating, inflammatory symptoms. This limits our ability to compare to previous work to interpret the differences observed by exposure time. The patent period is the time period most often investigated in parasitology as a factor that modifies host response and immunology (e.g., Jirků-Pomajbíková et al., 2018), but it is unlikely to be important here. Colitis was induced well into the patent period, which begins between 7 and 11 days post inoculation, in all experiments here (Figure 1) and also in other studies (Li et al., 2013; Defaye et al., 2018; Růžková et al., 2018). The density of *Blastocystis* is known to continue to increase over time (Chandramathi et al., 2014; Kumarasamy et al., 2017) and may be an important determinant of host response. *Blastocystis* is typically detected by culturing, a method also used here (for confirmation of colonization) that is accurate but does not give insight into the population size of *Blastocystis* in the gut, so this data is not generally available. Other research does show that the immune response to *Blastocystis* in rats is time dependent. For example, expression of TNFα, INFγ, and IL-12 is elevated 2 or 3 weeks after inoculation but does not differ from controls by 4 weeks post inoculation (Iguchi et al., 2009). Thus, we posit that the timeline of the *Blastocystis* colonization, and the timing of interventions and experimental observations following *Blastocystis* inoculation, is an important to *Blastocystis* colonization.

### Upregulation of IL17C/IL17re With *Blastocystis*

The elevated gene expression of IL17re/IL17C in *Blastocystis* colonized rats prior to colitis (Day 32) and 2 days after the induction of colitis (Day 88; Figure 4) is intriguing because this cytokine is reported to be involved in mucosal defense against pathogens and plays a role in mitigating the impacts of inflammation in models of induced colitis using dextran sulfate sodium (Ramirez-Carrozzi et al., 2011; Song et al., 2011; Reynolds et al., 2012). Expression of IL17re/IL17C is important in mucosal immunity against bacterial pathogens such as *Citrobacter* (Song et al., 2011), and this cytokine previously reported to be upregulated in response to infection by a variety organism, including viruses, fungi, and bacteria (Nies and Panzer, 2020). Our data suggest that IL17re/IL17C expression may be elevated in response to long-term *Blastocystis* ST3 colonization and induced in the response to colitis (Figure 4). Whether this plays a role in the faster recovery observed in *Blastocystis*-colonized rats is unknown. We only detected elevated IL17re/IL17C expression in the long-term experiment, and this difference may be related to exposure time. In the short-term experiment, the gene expression was at or below detection threshold and thus we did not obtain usable data.

### Influence of *Blastocystis* on the Microbiome

We reject the hypothesis that *Blastocystis* ST3 colonization leads to an increase in bacterial diversity in the gut, as we consistently find no difference in diversity over time or across experiments (Figure 7). This result is also consistent when we exclude bacteria found in the infectious dose from the calculation (as is the case for results presented in the main text), and when the bacteria in the infectious dose are retained. This is surprising as a number of observational surveys in asymptomatic human populations (Andersen et al., 2015; Andersen and Stensvold, 2016; Beghini et al., 2017; Forsell et al., 2017; Nieves-Ramirez et al., 2018; Tito et al., 2019) and experimental studies in rats (Defaye et al., 2020) show that bacterial diversity is elevated in the presence of *Blastocystis*. Host diet may be a cofounding variable in observational studies, as both *Blastocystis* presence and bacterial diversity are associated with diets higher in fiber and plant material (Forsell et al., 2017). Our results do suggest that long-term colonization of *Blastocystis* may mitigate bacterial diversity loss following colitis induction (Figure 7 and Table 2). This likely related to the faster recovery from intestinal inflammation and diarrhea, as we and others have previously noted precipitous declines in gut microbiome diversity during DNBS-induced colitis in rats (Jirků-Pomajbíková et al., 2018).

These data support the hypothesis that *Blastocystis* ST3 consistently alters the gut microbiota composition in rats and show that differences between control and *Blastocystis*-colonized rats are amplified with time and possibly with intestinal inflammation (Figure 7 and Table 2). Change in the community composition in the presence of *Blastocystis* infection has been consistently demonstrated from both experimental (Yason et al., 2019; Defaye et al., 2020) and observational studies (Nieves-Ramirez et al., 2018; Renelies-Hamilton et al., 2019; Tito et al., 2019; Gabrielli et al., 2020).

Although *Blastocystis* consistently altered the gut microbiome, the specific taxa that are enriched are depleted are largely experiment-specific and vary across days within each experiment (Figure 8 and Supplementary Figures 1–4). Previous studies have also reported different and conflicting suits of taxa enriched and depleted in the presence of *Blastocystis*. For instance, we did not observe a positive association between *Blastocystis* and the *Prevotella*-driven enterotype and a negative association with the *Bacteroides*-driven enterotype (as reported in Andersen et al., 2015). We did find consistent enrichment of *Bilophila* and *Butyricimonas* and depletion of Defluviitaleaceae in *Blastocystis*-colonized rats (Figure 8 and Supplementary Figures 1–4), potentially suggesting that they, or the functions they perform, have specific relationships with *Blastocystis* that could be investigated in further studies. We note that *Butyricimonas* was not detected prior to *Blastocystis* inoculation and in very few control samples. It may have been consistently found in the infectious dose even after washing, and despite bioinformatically removing all ASVs that were in the infectious dose samples.

We also note that after the induction of colitis, the bacterial communities associated with *Blastocystis* shifted in a more similar manner across experiments, becoming enriched with Clostridiales (Firmicutes) taxa and depleted in Bacteroidales (Bacteroidetes), while control rats showed the opposite pattern. These results hint that *Blastocystis* may prompt a similar response of the gut microbiome to intestinal inflammation, although this result contrasts with Defaye et al. (2020), which found that the ratio of Firmicutes to Bacteroidetes was lower in *Blastocystis* colonized rats. It is not clear what role, if any, the microbial changes observed play in the inflammatory response to *Blastocystis*. Ulcerative colitis patients have decreased abundance of *Butyricimonas* and *Bilophila* (Hirano et al., 2018; Salem et al., 2019) and a decrease in the ratio of Firmicutes to Bacteroidales (Kabeerdoss et al., 2015). Yet, these bacterial shifts cannot solely explain the protective effect since they were also observed in the short-term experiment. There are numerous differences in the bacteria enriched or depleted in the colonized rats across experiments (Figure 8 and Supplementary Figures 1–4), some of which may influence the immune response and faster recovery from colitis in the *Blastocystis* colonized rats. Further studies targeting key taxa, as well as inoculation controls of the bacteria from the infectious dose, are needed to elucidate the role bacterial changes associated with *Blastocystis* colonization play in health.

## Conclusion

Overall, our results suggest that long-term colonization of *Blastocystis* ST3 may be protective against intestinal inflammation by promoting faster recovery. These results may translate to humans, which are also colonized by *Blastocystis* for long time periods (Scanlan and Marchesi, 2008). Our results show that *Blastocystis* consistently alters the gut microbiome, but the patterns of taxa enriched and depleted vary across experiments, we find no change in microbiome diversity with *Blastocystis*. The one exception is that diversity of the *Blastocystis* group is maintained following colitis after long-term colonization, suggesting that *Blastocystis* may be associated with a stabilization of the gut ecosystem. Further studies are needed to verify whether the improvement in host health and the stabilization effect of microbiota persists beyond 2 days after colitis induction, and how repeatable this effect is for *Blastocystis* ST3 and for other subtypes. The results here may explain in part the enriched presence of *Blastocystis* in asymptomatic individuals compared to patients with IBS or IBD.

## Data Availability Statement

The data presented in the study are deposited in the European Nucleotide Archive repository, accession number PRJEB42752.

## Ethics Statement

The animal study, present experiments, and protocols were reviewed and approved by the Committee on the Ethics of Animal Experiments of the Biology Centre of the Czech Academy of Sciences (České Budějovice, Czechia, permit no. 33/2018) and by the Resort Committee of the Czech Academy of Sciences (Prague, Czechia).

## Author Contributions

ZL, OK, and MJ contributed to the experimental work. VB, ZL, MJ, LWP, and KJP contributed to the conceptualization. VB, ZL, OK, MJ, and LF contributed to the methodology. VB, ZL, MJ, LWP, and KJP contributed to the data curation. LWP and KJP contributed to the funding acquisition and project administration. VB, MJ, LWP, and KJP contributed to the software. VB, ZL, MJ, LWP, and KJP contributed to the validation. VB, ZL, MJ, LF, LWP, and KJP contributed to the draft writing. All authors contributed to the article and approved the submitted version.

## Conflict of Interest

The authors declare that the research was conducted in the absence of any commercial or financial relationships that could be construed as a potential conflict of interest.

## Footnotes

1 http://www.earthmicrobiome.org/protocols-and-standards/16s/

2 https://www.ebi.ac.uk/ena/browser

3 https://huttenhower.sph.harvard.edu/galaxy/

## Supplementary Material

Supplementary Figure 1 LEFSe cladograms depicting differentially abundant bacterial taxa associated with *Blastocystis* ST3 colonization in the long-term colonization (13 weeks). Day −7 is before *Blastocystis* ST3 colonization. Days 11, 21, 52, and 85 are after colonization and before colitis induction. Days 87 and 88 are after colitis induction. All days and sample types for which there was sufficient sample size and differentially abundant taxa between control and *Blastocystis* ST3 colonized groups. Each cladogram represents all taxa in the study. Yellow circles indicate no difference in relative abundance. Bacterial taxa significantly enriched in *Blastocystis* ST3-colonized group are in red (Kruskal-Wallis *P* < 0.05), while bacterial taxa enriched in the control are in green.

Supplementary Figure 2 LEFSe cladograms depicting differentially abundant bacterial associated with *Blastocystis* ST3 colonization in the short-term colonization (3 weeks). Days −1 and −7 are before *Blastocystis* ST3 colonization. Days 3, 9, and 20 are after colonization and before colitis induction. Days 22 and 23 are after colitis induction. All other notes are the same as Supplementary Figure 1.

Supplementary Figure 3 LEFSe cladograms depicting differentially abundant bacterial taxa associated with *Blastocystis* ST3 colonization in the short-term pilot study A. Days −25 and −7 are before *Blastocystis* ST3 colonization. Days 6 and 17 are after colonization. Colitis was not induced. All other notes are the same as Supplementary Figure 1.

Supplementary Figure 4 LEFSe cladograms depicting differentially abundant bacterial taxa associated with *Blastocystis* ST3 colonization in the long-term pilot study B. Day 1 is before *Blastocystis* ST3 colonization. Days 15, 36, and 50 are after colonization and before colitis induction. There is insufficient sample size for LEFSe analysis after colitis induction (Day 99) and it is not shown. All other notes are the same as Supplementary Figure 1.

Supplementary Figure 5 Effect of *Blastocystis* ST3 colonization on rat gut bacterial diversity in the short-term Pilot study A and the long-term Pilot study B. NMDS plots of Bray-Curtis dissimilarity visualize the difference in gut microbiota composition from fecal samples between the control group (warm shades) and rats colonized by *Blastocystis* ST3 (cool shades). **(A)** Short-term Pilot study A before *Blastocystis* ST3 colonization. **(B)** Long-term Pilot study B before *Blastocystis* ST3 colonization. **(C)** Short-term Pilot study Aafter *Blastocystis* ST3 colonization. **(D)** Long-term Pilot study Bafter *Blastocystis* ST3 colonization. Differences between groups are calculated with a PERMANOVA on combined days (those shown in each panel) followed by Benjamini-Hochberg correction.
