# Supplementary figures and images for: Blastocystis Colonization Alters the Gut Microbiome and, in Some Cases, Promotes Faster Recovery From Induced Colitis
Source: Front Microbiol. 2021 Apr 7;12:641483. doi: 10.3389/fmicb.2021.641483 (PMC8058373; doi:10.3389/fmicb.2021.641483)

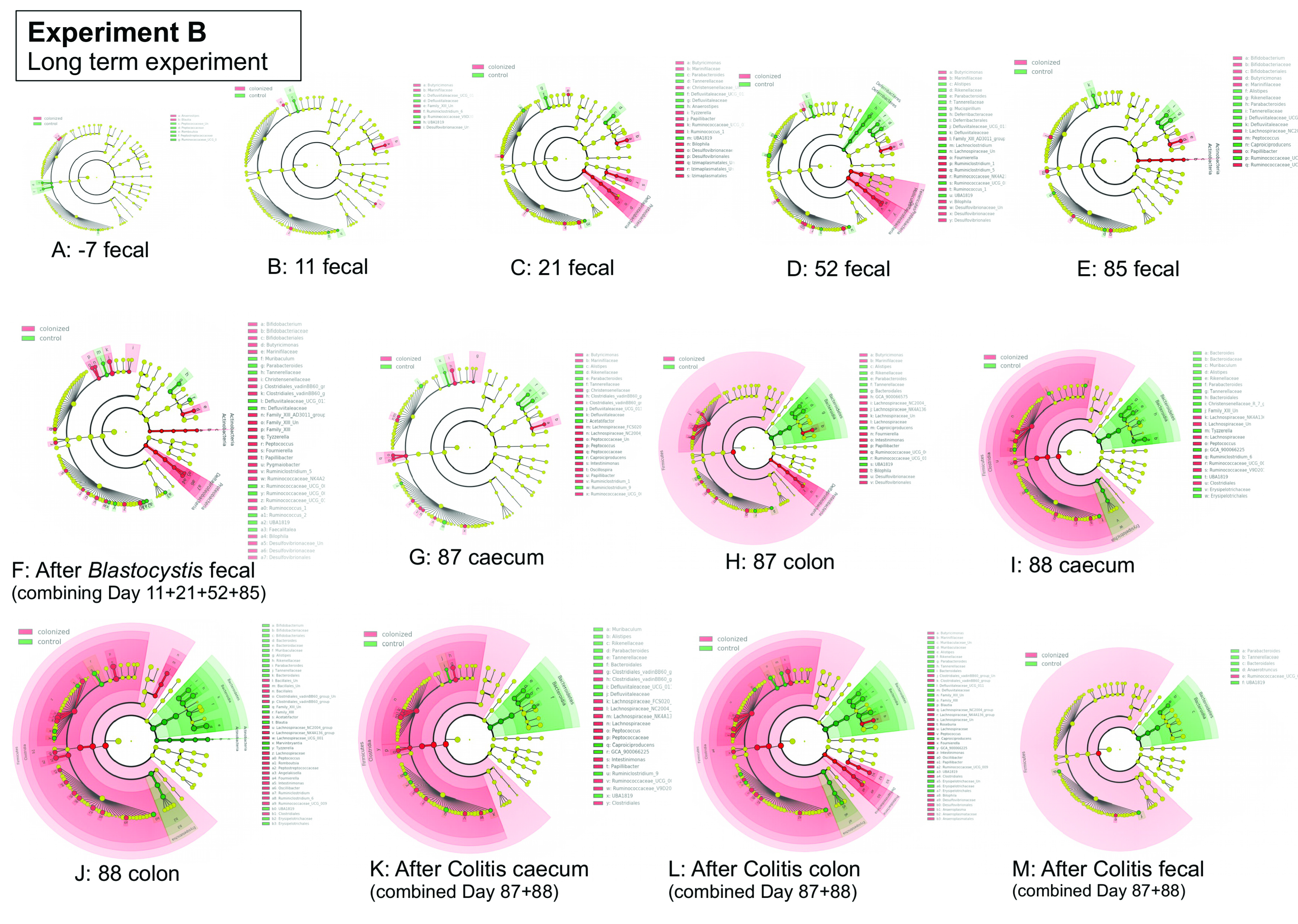

Supplement: Supplementary Figure 1 — LEFSe cladograms depicting differentially abundant bacterial taxa associated with Blastocystis ST3 colonization in the long-term colonization (13 weeks). Day −7 is before Blastocystis ST3 colonization. Days 11, 21, 52, and 85 are after colonization and before colitis induction. Days 87 and 88 are after colitis induction. All days and sample types for which there was sufficient sample size and differentially abundant taxa between control and Blastocystis ST3 colonized groups. Each cladogram represents all taxa in the study. Yellow circles indicate no difference in relative abundance. Bacterial taxa significantly enriched in Blastocystis ST3-colonized group are in red (Kruskal-Wallis P < 0.05), while bacterial taxa enriched in the control are in green. [file Image_1.JPEG]

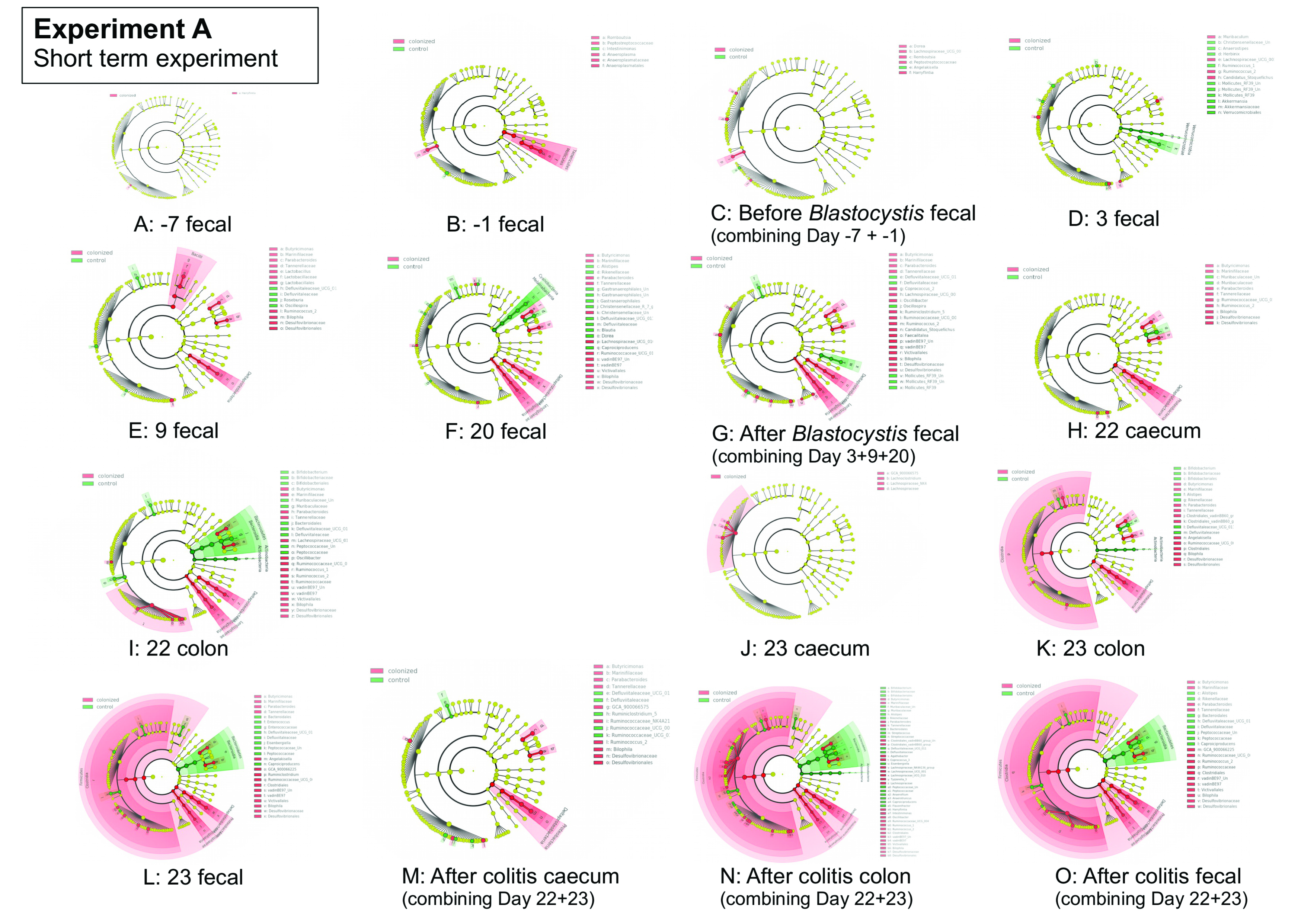

Supplement: Supplementary Figure 2 — LEFSe cladograms depicting differentially abundant bacterial associated with Blastocystis ST3 colonization in the short-term colonization (3 weeks). Days −1 and −7 are before Blastocystis ST3 colonization. Days 3, 9, and 20 are after colonization and before colitis induction. Days 22 and 23 are after colitis induction. All other notes are the same as Supplementary Figure 1. [file Image_2.JPEG]

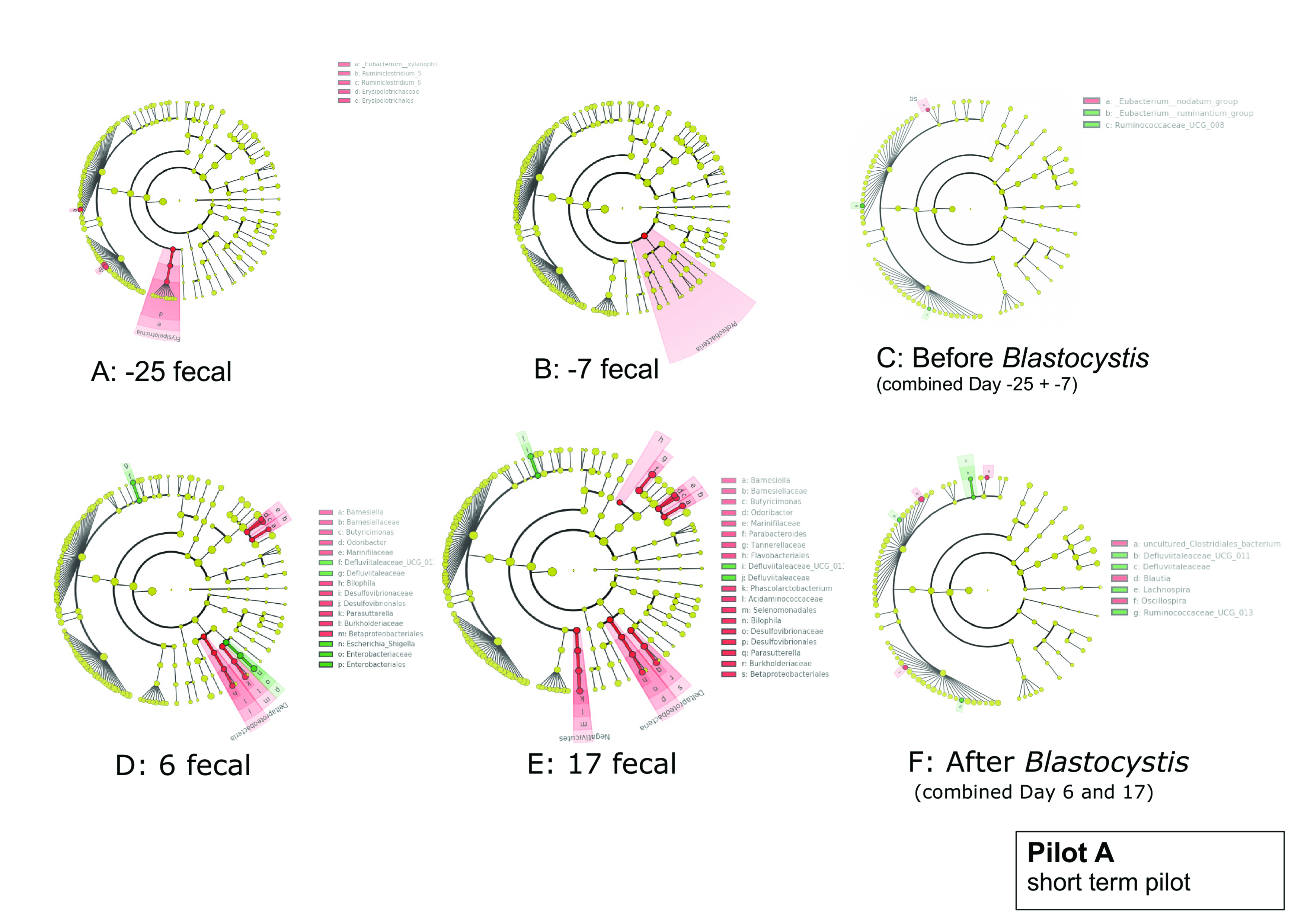

Supplement: Supplementary Figure 3 — LEFSe cladograms depicting differentially abundant bacterial taxa associated with Blastocystis ST3 colonization in the short-term pilot study A. Days −25 and −7 are before Blastocystis ST3 colonization. Days 6 and 17 are after colonization. Colitis was not induced. All other notes are the same as Supplementary Figure 1. [file Image_3.jpg]

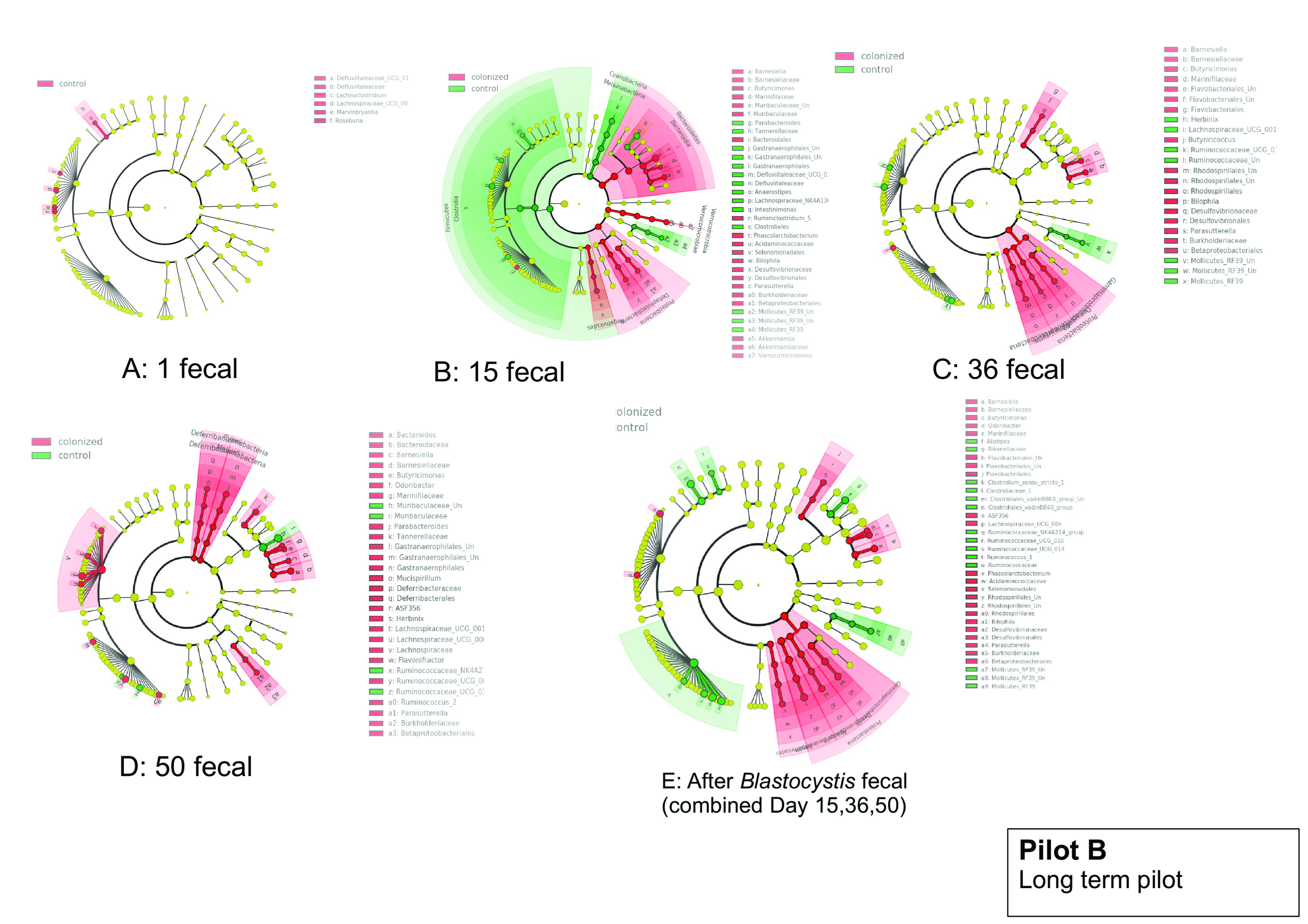

Supplement: Supplementary Figure 4 — LEFSe cladograms depicting differentially abundant bacterial taxa associated with Blastocystis ST3 colonization in the long-term pilot study B. Day 1 is before Blastocystis ST3 colonization. Days 15, 36, and 50 are after colonization and before colitis induction. There is insufficient sample size for LEFSe analysis after colitis induction (Day 99) and it is not shown. All other notes are the same as Supplementary Figure 1. [file Image_4.JPEG]

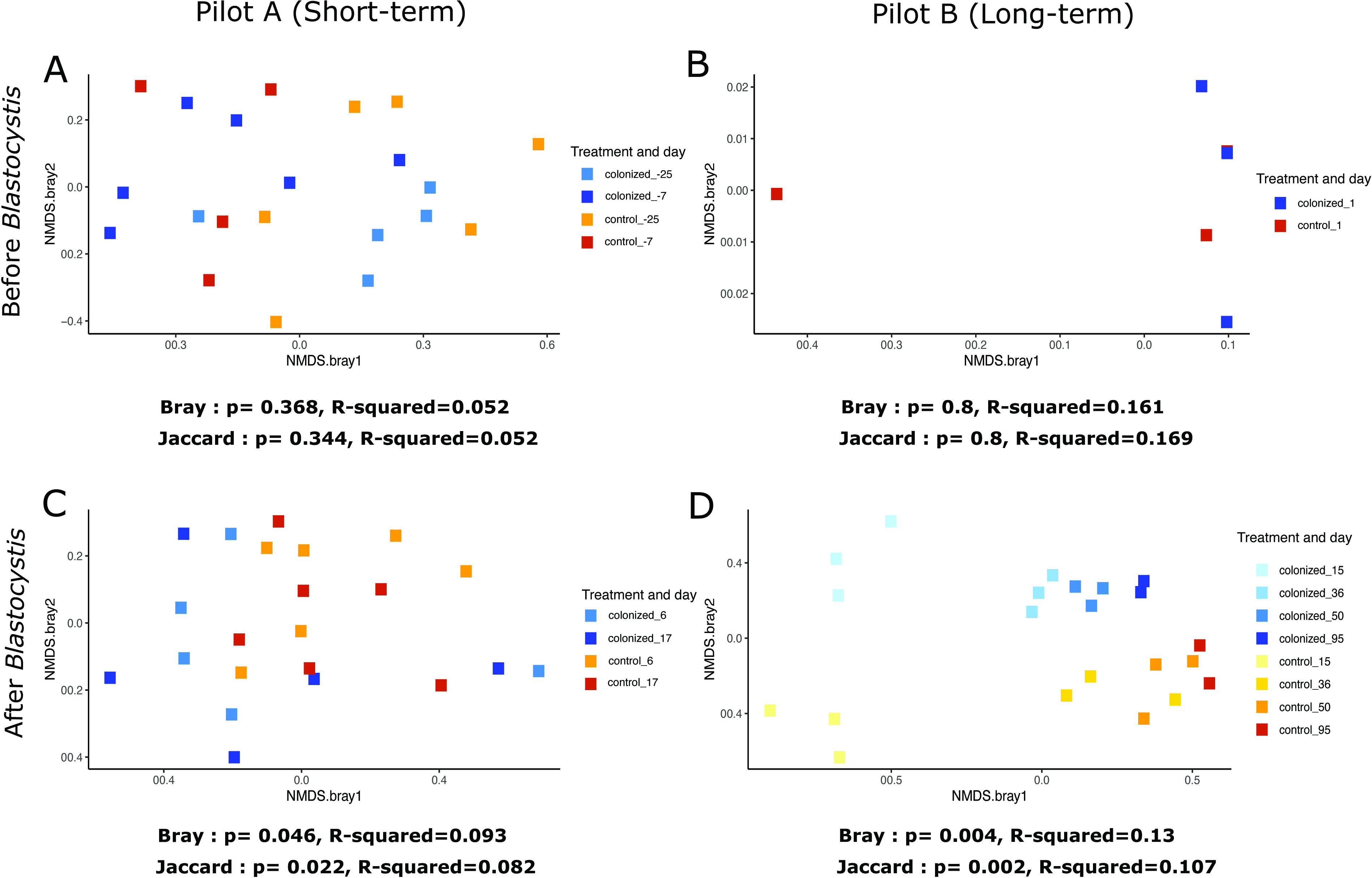

Supplement: Supplementary Figure 5 — Effect of Blastocystis ST3 colonization on rat gut bacterial diversity in the short-term Pilot study A and the long-term Pilot study B. NMDS plots of Bray-Curtis dissimilarity visualize the difference in gut microbiota composition from fecal samples between the control group (warm shades) and rats colonized by Blastocystis ST3 (cool shades). (A) Short-term Pilot study A before Blastocystis ST3 colonization. (B) Long-term Pilot study B before Blastocystis ST3 colonization. (C) Short-term Pilot study Aafter Blastocystis ST3 colonization. (D) Long-term Pilot study Bafter Blastocystis ST3 colonization. Differences between groups are calculated with a PERMANOVA on combined days (those shown in each panel) followed by Benjamini-Hochberg correction. [file Image_5.jpg]
